# Polydimethylsiloxane Composites Characterization and Its Applications: A Review

**DOI:** 10.3390/polym13234258

**Published:** 2021-12-05

**Authors:** Ronaldo Ariati, Flaminio Sales, Andrews Souza, Rui A. Lima, João Ribeiro

**Affiliations:** 1 ESTiG, Instituto Politécnico de Bragança, 5300-252 Bragança, Portugal; a46685@alunos.ipb.pt (R.A.); fcflaminio@gmail.com (F.S.); jribeiro@ipb.pt (J.R.); 2 MEtRICs, Mechanical Engineering Department, Campus de Azurém, University of Minho, 4800-058 Guimarães, Portugal; andrewsv81@gmail.com; 3 CEFT, Faculdade de Engenharia da Universidade do Porto (FEUP), Rua Roberto Frias, 4200-465 Porto, Portugal; 4 CIMO, Instituto Politécnico de Bragança, 5300-252 Bragança, Portugal

**Keywords:** PDMS, PDMS composites, mechanical properties, optical properties, biocompatibility

## Abstract

Polydimethylsiloxane (PDMS) is one of the most promising elastomers due its remarkable proprieties such as good thermal stability, biocompatibility, corrosion resistance, flexibility, low cost, ease of use, chemically inertia, hyperplastic characteristics, and gas permeability. Thus, it can be used in areas such as microfluidic systems, biomedical devices, electronic components, membranes for filtering and pervaporation, sensors, and coatings. Although pure PDMS has low mechanical properties, such as low modulus of elasticity and strength, it can be improved by mixing the PDMS with other polymers and by adding particles or reinforcements. Fiber-reinforced PDMS has proved to be a good alternative to manufacturing flexible displays, batteries, wearable devices, tactile sensors, and energy harvesting systems. PDMS and particulates are often used in the separation of liquids from wastewater by means of porosity followed by hydrophobicity. Waxes such as beeswax and paraffin have proved to be materials capable of improving properties such as the hydrophobic, corrosion-resistant, thermal, and optical properties of PDMS. Finally, when blended with polymers such as poly (vinyl chloride-co-vinyl acetate), PDMS becomes a highly efficient alternative for membrane separation applications. However, to the best of our knowledge there are few works dedicated to the review and comparison of different PDMS composites. Hence, this review will be focused on PDMS composites, their respective applications, and properties. Generally, the combination of elastomer with fibers, particles, waxes, polymers, and others it will be discussed, with the aim of producing a review that demonstrates the wide applications of this material and how tailored characteristics can be reached for custom applications.

## 1. Introduction

Polymers are a large class of materials that are widely used and the basis of several industrial goods [1]. They are formed by means of the chemical linking of smaller molecules (the monomers), and according to the number and characteristics of these bonded molecules, different properties can be achieved [2]. Natural polymers or biopolymers are those obtained from nature, for example, polysaccharides and proteins [3]. Synthetic or artificial polymers are produced in laboratories and are mostly derived from petroleum, e.g., acrylic, polyvinyl chloride (PVC), polyethylene, and polypropylene. Among the polymers are elastomers that have viscous and elastic characteristics. It is considered a viscous substance and has a dense, thick, and sticky consistency. Its applications are selected for materials that, when tension is applied, have the property of returning to their original shape, such as tires, rubbers, and elastics [4]. Polydimethylsiloxane, commonly known as PDMS, is a component belonging to the popularly known organosilicon group of silicones. PDMS is the most widely used silicone based on organic polymers that presents the characteristics of a transparent, flexible compound with biocompatibility properties [5]. As such, they are used in various applications and can be found in daily use in products such as synthetic fibers, plastic bags, paints, lenses, glues, and biomedical devices [6,7,8,9,10,11]. In addition, the polymeric industry has grown quickly and nowadays is one of the biggest industries [12].

An important subclass of polymeric materials is the elastomers. They are cross-linked polymers that have, in general, a low Young’s modulus, high-yield strains, and the ability to restore the original shape when a stress ceases. Examples of elastomers include natural and synthetic rubbers, silicone elastomers, and other copolymers [13,14].

**Figure 1:**
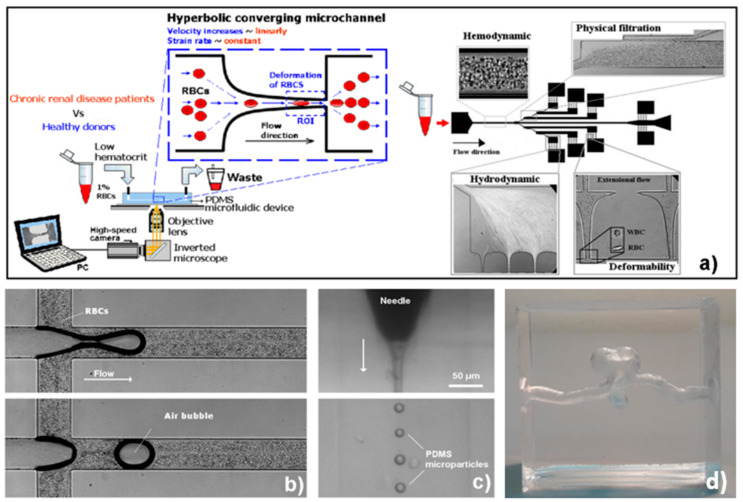
PDMS applications: (**a**) in microfluidic devices to assess motions and deformations of red blood cells (RBCs) from healthy donors and pathological patients [36]; (**b**) in microchannel networks to investigate gas embolism [38]; (**c**) with a flow-focusing technique to generate micro-sized PDMS particles [44]; (**d**) with PDMS biomodels to assess the blood flow behavior in aneurisms [39].

One of the most promising elastomers is the polydimethylsiloxane (PDMS). It is a highly used silicon-based polymer with good chemical and thermal stability [4,15,16,17], biocompatibility [18,19,20], corrosion resistance [21,22], flexibility [23,24,25], repeatability [26], a low cost [27], ease of use, chemically inertia, hyperplastic characteristics, and gas permeability [3,28]. Thus, PDMS has been used in several fields and systems (see Figure 1), such as microfluidics and nanofluidics [29,30,31,32,33,34,35,36,37], biomodels [38,39], organ-on-a-chip platforms [40,41], blood analogues [42,43,44,45,46], electronic components [47], membranes for filtering and pervaporation [48,49,50], sensors [51,52,53], optical and thermal devices [54,55,56], coatings [57,58,59], etc. However, several PDMS applications can be compromised due to its low mechanical properties, such as low elasticity modulus and strength. One way to overcome this limitation is by performing bulk modifications that create PDMS composites with tailored properties. These modifications can be achieved by inserting free molecules (nano or microparticles) and by changing the pre-polymer composition [25]. In addition, Mi et al. [43] have shown that the tensile properties of PDMS can be improved by adding silica fibers.

Despite the large number of papers that show the applications and properties of pure PDMS, there are few works dedicated to listing, reviewing, and comparing information about its composites. Hence, this review shows not only the PDMS composites’ characterization and their applications but also the potential of this material. First, an overview of the research studies performed with PDMS composites was made, highlighting the works where the addition of other materials in PDMS has promoted significant changes in PDMS’s properties. This review is focused on discussing the combination of PDMS with fibers, particles, waxes, and polymers, among others, and its potential applications.

## 2. Polydimethylsiloxane

PDMS is a polymer classified as a silicon elastomer, which means it is constituted by a combination of inorganic chains with high surface energy, associated with silicates, methyl groups, inorganics, and low surface energy. Inside PDMS’s chemical structure, these methyl groups are prevalent and crucial to provide PDMS hydrophobic characteristics, with a surface tension around 20.4 mN/m [60].

The synthesis of PDMS typically begins with hydrolysis and the condensation reaction of dichlorosilanes, obtaining cyclic and linear polymers [61]. This synthesis methodology results in a weak control of molecular weight originating polymers with low properties, which cannot be used in most practical applications [62]. Hence, to improve the control of the molecular parameters, the previous synthesis method was progressively replaced by the ring-opening polymerization of cyclic siloxanes. It should be noted that the kinetically controlled polymerization of PDMS is based on the anionic polymerization of hexamethylcyclotrisiloxane monomer. This synthesis method allows the formulation of almost monodisperse PDMS with customized structures based on a chain reaction in which a specific catalyst reacts with hexamethylcyclotrisiloxane to generate short silanolate-ended chains that are able to attack other hexamethylcyclotrisiloxane molecules to produce the desired polymer [63].

The chemical structure consists of a (Si-O) backbone and repetitive (Si (CH_3_)_2_ O) units that can be expressed as CH_3_[Si (CH_3_)_2_ O]*_n_* Si (CH_3_)_3_, whereas *n* represents the number of repetitive units. These repetitions define materials’ molecular weight, which consequently defines properties such as viscoelasticity. Furthermore, (CH_3_) represents the methyl group, and (Si–O) represents the strength of the siloxane bonds that make this material chemically and thermally stable. The crosslinking reaction, with groups such as phenyl and vinyl, can carry out large property changes for different applications. Crosslinking reactions, with groups as phenyl and vinyl can accomplish great property changes for different applications [25,64,65].

One of the main areas where this elastomer is used is in the biomedical field; thus, the material presents high biocompatibility and biostability. These terms are related to materials that do not cause adverse effects when they come into contact with biological tissues. Although the mechanism for biocompatibility is still not totally clearly demonstrated, it is known that interactions with water in proteins are fundamental parameters and are related to physicochemical characteristics such as surface free energy, stiffness, surface charge, and wettability [66].

Another important aspect of biomaterials refers to structural biocompatibility. This is related to the mechanical interactions between an implanted device and the surrounding tissues. The mismatch of mechanical properties can cause inflammation or incorrect support of the efforts present [67].

Regarding mechanical properties, pure PDMS usually shows an elastic modulus between 1.32 and 2.97 MPa and tensile strength from 3.51 to 5.13 MPa. These values vary depending on the curing agent ratio and curing temperature during the manufacturing process. The tensile values rise with the increasing temperature until it reaches 125 °C; temperatures above this value reduce the tensile strength, but Young’s modulus continues growing and presents a linear relation with the temperature. [68,69]. An increase in the curing agent ratio can lead a reduction in PDMS’s flexibility and, consequently, reduces the Young’s Modulus [70,71]. The hardness is usually proportional to the Young’s modulus and presents values around 43 Shore A [72,73,74].

Besides being optically transparent, having a low cost and high capability to replicate models as well as its use in rapid prototyping, pure PDMS has limitations. For instance, high hydrophobicity can be a problem when filling microchannels, as it makes it necessary to resort to temporary surface treatment procedures, such as oxygen plasma. Other restraints occur due to the permeability of the material, which can interfere with cell cultures. The main disadvantage of PDMS is its structural application, which may be extremely specific and reduced. Furthermore, the modification of its characteristics, such as transparency, can be interesting for the use in sensors and some types of coatings [25,67,75].

## 3. PDMS Composites

Despite the large deformation capacity of the pure elastomers, fillers or reinforcements are often used to create composites that usually exhibit increased stiffness modulus, fracture toughness, fatigue resistance, tensile strength, and abrasion resistance [76,77].

Thus, filler characteristics such as volumetric fraction, shape, size, and dispersion are extremely important [78].

**Figure 2:**
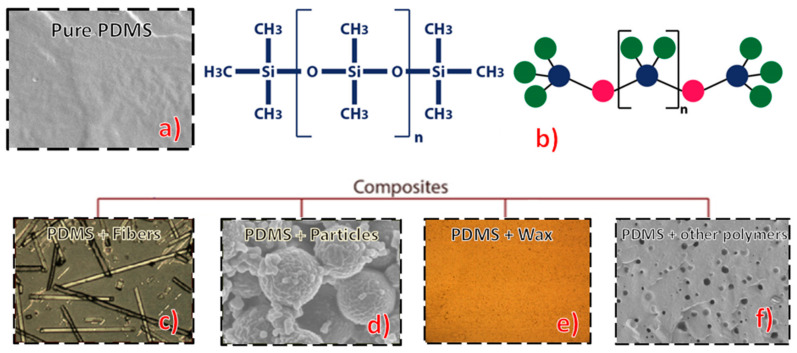
Pure PDMS and its compounds discussed in the present work: (**a**) Pure PDMS; (**b**) CH_3_[Si (CH_3_)_2_O] *n* Si (CH_3_)_3_; Composites: (**c**) with fiberglass reinforcement, adapted image from [81], (**d**) with tantalum ethoxide-nanoparticles, adapted image from [82], (**e**) with beeswax (**f**) with other polymer combination polyethylene glycol (PEG), image adapted from [83].

Even more crucial for the final properties is the interaction between the elastomer and the filler, which increases the degree of crosslinking. This characteristic is optimized if there are reactive surface groups and the particles inserted are small, which is the reason why many of the reinforcements used in this type of composite are nano- or microfibers and particles (see Figure 2a–d). Additionally, some agents can help the dispersion and coupling of the fillers in the composite. Usually, they are bifunctional molecules that provide bridges at the polymer–filler interface. Examples are silicone coupling agents, mercaptopropyltrimethoxysilane, and (3-triethoxysilylpropyl) tetra sulfide [79,80].

Therefore, the combination of PDMS with other materials allows the optimization and expansion of its applications and has already been extensively explored. The next topics discuss some of the main composites of the PDMS matrix, dividing them into materials filled with fibers or particles, and materials in which there is a combination of waxes (Figure 2e), polymers (Figure 2f), or another additive.

### 3.1. Fiber- and Nanofiber-Reinforced PDMS

Fiber-reinforced composites are one of the most successful materials in engineering applications nowadays. Besides the fact that polymers such as epoxy or polyurethane usually comprise the matrix, the use of elastomer matrices is promising for applications such as a “muscle” actuator, flexible surfaces for aircraft aerodynamic components, and safer flywheels [84]. Additionally, for biomedical applications, carbon fillers inside a polymer can become an alternative to metallic devices, as they present advantages such as radiolucency and high mechanical properties. However, after cyclical sterilization, the interaction between phases can be compromised [85].

Likewise, fibrous fillers for PDMS are not widely studied in the literature. The distribution of reinforcements can be difficult, especially when using nanofibers, generating fillers’ agglomeration and voids, which impairs and complicates their mechanical properties. However, studies in which electrospun polyacrylonitrile-graft-polydimethylsiloxane fibers were introduced as a graft copolymer on a PDMS matrix in non-woven and aligned portions reached satisfactory results, increasing the tensile strength from 0.3 MPa up to 2.3 MPa. Additionally, the Young Modulus, which was 47 MPa for the pure silicone sample, increased to 119 MPa when using non-woven fillers and 674 MPa for aligned reinforcements [86]. Another work reached an increase in mechanical and thermal properties when using a PDMS and graphene foam matrix reinforced with carbon fiber. The increase in the tensile strength, Young’s modulus, and thermal conductivity was 52%, 71%, and 41%, respectively, when comparing it with the pure matrix [87].

A combination of PDMS with carbon fillers has already been proposed as an alternative for the fabrication of flexible displays, batteries, wearable devices, and tactile sensors used for robotics [49,50]. By combining short carbon fiber with elastomer in a method known as spatial confining forced network assembly, an electrical conductivity around 1.67 × 10^2^ S/m was obtained, which is superior to that presented by pure PDMS, an insulator with conductivity in the order of 10^−12^ S/m. Additionally, when pre-compressed, this composite can reach even higher conductivity, around 3.2 × 10^2^ S/m [88].

When using dual-scale carbon fillers, a combination of carbon nanofibers and short carbon fibers, the disparity between the elastic modulus of the elastomeric matrix and the conductive graft, used as a sensor, was reduced. This leads to the increased sensitivity, stretchability, and repeatability of high-strain sensors, reaching small drifts in resistance even after 300 loading cycles [89]. A related application can be found in the manufacturing of harvesting energy devices. These are normally constituted by piezoelectric ceramic and present problems such as the possibility of failures when submitted to cyclical efforts, due to the fragility of the ceramic, and environmental impact, as they usually contain lead, which is classified as a substance of very high concern. K_0.485_Na_0.485_Li_0.03_NbO_3_ (KNLN) fibers in PDMS have achieved a piezoelectric charge and voltage comparable to that of ceramic PZTs in a mechanically compliant and environmentally friendly material [90]. Satisfactory results for energy harvesting and piezoelectric uses were also assessed using the elastomeric matrix and BaTiO_3_ nanofibers, another lead-free alternative that can be aligned in the matrix and reached output voltage values between 0.56 and 2.67 V when a periodic mechanical compression with a pressure of 2 Pa was applied [91]. Additionally, when using silver microfibers in a soft litography process, the conductivity of the PDMS increased, changing the resistance from 0.12 Ωm (for 10%wt) to 0.000001 Ωm; using 8%wt of short carbon fiber fillers, the resistance of a 0.1-millimeter-thick sample was 0.0026 and could reach values three times lower with the insertion 1%wt of a second conductive nanofiller [92,93].

Reinforcements can also allow the use of PDMS in high-performance composites, maintaining good transparency. Studies using just 1%wt of three-dimensional silica continuous fiber reinforcements were achieved, in comparison with the pure matrix, which showed increases of 140%, 94%, 18%, and 95% in tensile modulus, strength, maximum strain, and tear strength, respectively [81]. The optical properties of the elastomer can also be explored to analyze fiber alignment in processes such as injection molding, where a study was able to visualize the flow during the injection and determine how carbon fiber inserts’ positioning could be improved [94]. Furthermore, the use of PDMS as an NCF fiber-optic interferometer coating showed excellent thermal performance, resulting in a significant improvement in temperature sensitivity when compared to a pure silica fiber interferometer. PDMS was used as fiber coating to improve the fragility of the fiber structure, thus increasing its reliability for practical applications [53]. Likewise, tailored properties can be achieved to match living tissue requirements, such as a bone elasticity modulus. This way, the combination of 55 vol.% of aramid balanced fabric reinforcement, PDMS, and 0–25% vol.% nano/micro hydroxyapatite and tricalcium phosphate reached a modulus of elasticity, in bending, similar to that presented by human cortical bones (14–20 GPa), which is suitable for bone surgeries [95]. For stretchable electronics, a PDMS/plain weft-knitted nylon fabric showed a huge increase in fracture toughness of about 700%, which is much higher than that of pure PDMS, and maintaining high elasticity (Young’s modulus around 2 MPa). Additionally, during cyclic loading, for small stretches, the behavior was linear elastic, and, for a relatively large stretch, it had significant hysteresis [96].

### 3.2. Addition of Particles to PDMS

Particles are one of the most common additions in PDMS. When one of the dimensions of the inserted fillers is smaller than 100 nm, the formation of nanocomposites occurs. Another common occurrence is micro composites, with particles on the micrometric scale. Examples of particles include carbon nanotubes, some silicates, and graphene [25].

**Figure 3:**
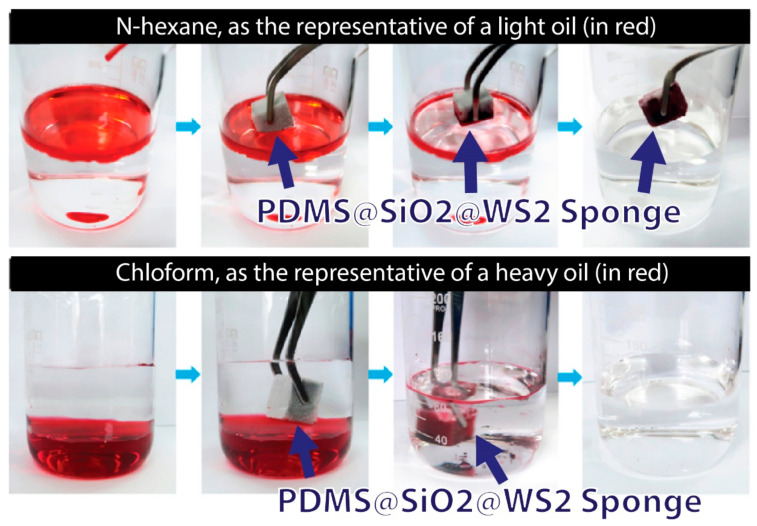
PDMS/SiO_2_/WS_2_ sponge for application in oil separation, adapted from [97].

Porous compounds using PDMS and particulates are commonly used to improve absorption efficiency in the separation of liquids from wastewater due to their porosity and super hydrophobicity. PDMS shows benefits for the reusable compound, as it can be pressed repeatedly without structure loss. Additionally, as a low-cost material, it can reduce the cost of effluent treatment, as these processes generally require a large number of absorbent materials. Nanoparticles are used to improve surface roughness, which leads to an increase in super hydrophobicity, reaching a contact angle with water greater than 150° and allowing the droplets to slide under the surface. PDMS acts as a strong binding and immunization adhesive for nanoparticles, and one of the main advantages of this application is its non-toxicity. Some works manufactured porous compounds modified with SiO_2_ nanoparticles and others used TiO_2_ nanoparticles as modification agents, coated with PDMS and manufactured by a simple immersion technique. Melanin sponges have high efficiency in removing oil from water through micro- and nanoparticles of tungsten disulfide (WS_2_) and SiO_2_, respectively. The particles are immobilized on the sponge surface by a layer of polydimethylsiloxane adhesive that features an extremely water-repellent structure by a simple one-step immersion process. The composite sponge has high oil absorption at 21e112 times its own weight and a selectivity efficiency above 99.8%, as shown in Figure 3 [97]. Likewise, using silicon nanoparticles as a coating substrate was produced by simple immersion followed by magnetic stirring in order to reduce the surface drag force. The drag force reduction rate decreased by 24%, and the coating showed greater durability in acidic and alkaline solutions [98]. TiO_2_ additives are also applied to create electroactive material based on PDMS; this material shows reduced drive voltage and response speed in comparison with a traditional silicone-poly(hexylthiophene) electroactive polymer [99].

Metallic meshes with different pore sizes were coated with nanoparticles and nanocomposites by an immersion process. Hydrophobic titanium diode was used as a nanoparticle and polydimethylsiloxane as a binding resin. The presence of PDMS resulted in an improvement in the mechanical durability of wire mesh. Besides that, it was found that meshes with smaller pores are more efficient in separation; however, the process takes longer [100]. Likewise, a porous sponge with photocatalytic properties, manufactured with PDMS and TiO_2_, was used for the degradation and demolition of organic pollutants in textile wastewater. These nanoparticles were injected into the polydimethylsiloxane sponge, were able to demote up to 50% of the pollutants without the presence of light and 80% with light; this is due to degradation of the photocatalytic action of TiO_2_, allowing greater absorption of the dye from the solution [101]. Furthermore, TiO_2_ nanoparticles were added to the PDMS matrix by a spin-coating method and used as coatings on metals to improve the corrosive capacity of the material. Corrosion resistance improved with the 8% by weight proportion of TiO_2_, which achieved the best performance [102].

In addition, metallic particles in PDMS films or substrates have been studied for various applications. For example, micro-pumps can be created, taking advantage of the elasticity and mobility of PDMS membranes and using magnetic actuators to generate deflections by attracting iron particles [103]. The same composite with magnetic properties could find application in the control of droplet motion, a useful resource in the manipulation of sample drops in chemical and biological tests and studies [104]. When comparing different magnetic fillers, pristine carbonyl iron microparticles (CI) reached a maximum deflection of 762 μm at 0.27 mT for a membrane diameter of 6.2 mm, which was the highest result in a study that also evaluated lauric acid-coated superparamagnetic iron-oxide nanoparticles (SPION-LA) and lauric acid-coated carbonyl iron microparticles (CI-LA) [105].

The porosity and water absorption of the PDMS cell scaffolds are inversely proportional to particle size. However, the interconnectivity of PDMS cell scaffolds increases with increasing particle dimension. Additionally, mechanical properties such as compressive modulus and compressive strength obtained higher values for intermediate pore sizes, between 300–450 µm, in a sample universe with 150–300, 300–450, and 450–600 µm pores [106].

### 3.3. Wax Addition

Waxes such as beeswax and paraffin proved to be materials capable of improving the hydrophobicity corrosion resistance and the thermal and optical properties of PDMS, leading to applications such as wearable devices, sensors, and superhydrophobic coating [75].

A phase change functional compound consisting of PDMS such as matrix and paraffin was prepared by the molten mixture method and has demonstrated excellent performance in thermal and mechanical properties. The compound has excellent flexibility and heat absorption capacity. As the paraffin content increases, the temperature sensitivity of the mechanical property also increases, and the composite’s storage modulus decreases with increasing temperature. Consequently, substrate thermal management flexibility is enhanced with increasing temperature, thus applying to a flexible substrate. The change in the transmittance of composites due to the phase change of the paraffin makes it applicable as a visual warning of temperature increase [107]. Additionally, with the addition of paraffin spheres in the PDMS matrix, the composite had an increase of more than an order of magnitude in stiffness in a compression test [108]. Another work aimed to improve the transparency of the compound with a percentage of up to 10%wt of paraffin without changing the transparency of the film. During optical testing, the compound was determined not to be ideal for use as a transparent reflective key, but there have since been considerable additional improvements [109].

**Figure 4:**
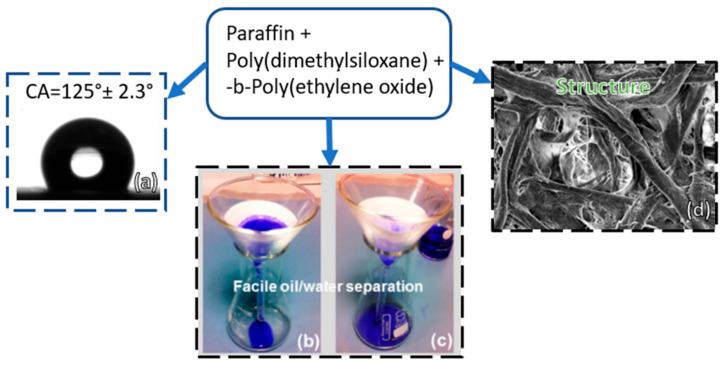
Oil–water separation by filter paper, oil remains on the filter paper while water with blue dye passes through the filter paper, adapted from [111]: (**a**) Contact angle, (**b**,**c**) oil/water separation and (**d**) structure of filter paper.

Another interesting coating using PDMS combined with paraffin wax is found in the textile engineering field, where it is manufactured using a simple inlaying process. The prepared textiles exhibited stability after mechanical abrasion and chemical corrosion. Furthermore, the coated textiles have excellent self-cleaning and water repellence abilities and can be used to separate various types of liquid mixtures such as oil–water, diesel oil, and crude oil. The separation efficiency can reach up to 95% in the separation of the diesel oil–water mixture specifically [110]. A new method of modifying the surface properties of filter papers, making them super hydrophilic and super oleophobic underwater in a simple and economical way, was presented in Figure 4. Both super hydrophilic and super oleophobic paper can reach up to 99% efficiency for gravity oil–water separation [111].

Other transparent and flexible composite films with a selectable mist using a lamination process have been manufactured. The film is composed of polyethylene terephthalate, graphene, and paraffin organogel-PDMS substrates. When graphene is heated, a transformation occurs in the paraffin impregnated in the PDMS, leading to an improvement in light scattering. Transmittance is maintained above 90% in the visible range, while fog can be controlled in the range from 0.5 to 85% by applying a voltage of 18 V with a consumption of 0.33 W/cm^2^. Optical film that enables transmittance control can be used to enhance the light-capturing properties of photovoltaic panels and windows, allowing for privacy [112]. Another method consists of applying a voltage of 10 V on the composite film, which then undergoes a rapid change from opaque to transparent in less than 8 s followed by transmittance, which changes from 2% to 75% [113].

However, it is also possible to have adhesive films with the same function of switching between opaqueness and transparency via a thermal trigger. The appearance of the film alternates between opaque at room temperature and clear at temperatures above 53 °C. The change in optical properties is almost instantaneous, and the application is best suited for use as a low-cost, smart window coating [114].

In order to meet the needs of protection against oxidative substances, corrosive liquids and ultraviolet light that limit the practical application of solar vapor generation were employed. Photothermal conversion coatings were manufactured with stable chemical and mechanical properties by a spray process with a mixture of beeswax, multiwalled carbon nanotubes, and polydimethylsiloxane. The coatings exhibited good broadband light absorption capacity efficiently under sunlight irradiation. The superhydrophobicity caused by beeswax and PDMS provides self-cleaning that can prevent the reduction in steam generation efficiency induced by microorganisms and mud in the water and are able to heal damage to superhydrophobicity through the migration of beeswax, providing lasting protection. Due to their low maintenance requirements, simple preparation process and high cost effectiveness, photothermal conversion coatings are suitable for supplying fresh water to remote or disastrous areas [115].

Another work used natural carnauba wax and PDMS to fabricate superhydrophobic surfaces using model transfer and colloidal deposition method, which consists of depositing carnauba wax on the surface. The secret to the superhydrophobic surface was rubbing to remove the weakly connected wax particles. After removing the loosely bound particles, the surface becomes super-hydrophobic with a contact angle greater than 150° and a slip angle less than 10°. The surface has good mechanical durability against abrasion and water impact. As they are biocompatible materials and possess these characteristics, they have become widely used in biomedical applications due to their repellency to blood and its components, presenting reduced drag to the blood and a coagulation time of at least one hour [116]. This makes it ideal for manufacturing blood-compatible materials and microfluid devices used in blood separation and typing as well as its application to self-cleaning surgical garments. The super hydrophobic surface containing carnauba wax and PDMS has good mechanical durability against abrasion and water impact and is therefore suitable for outdoor environments [117].

Another application of PDMS combined with paraffin wax is used in a new hermetic encapsulation method for microfluidic devices actuated by negative pressure. The airtight materials used are non-active, non-hazardous, and commonly used as sealing materials for plastic medicine and food packaging. The new method offers advantages such as the ability to steer for more than 3 weeks without any vacuum equipment and only allowing air intake when the device is encapsulated [118].

### 3.4. Blends with Other Polymers

The search for ecological materials for membrane separation is becoming highly attractive and increasingly competitive, always focusing on the low cost and high separation efficiency that are present in the main industrial processes. New gas separation membranes using a copolymer of poly (vinyl chloride-*co*-vinylacetate) (PVCA) and polydimethylsiloxane (PDMS) were manufactured using a simple method of mixing followed by constant agitation. The separation performance was carried out by permeation studies on pure phases CO_2_, N_2_, and CH_4_ at different temperatures. After characterization tests, the membranes showed better selectivity and high flux at 25 °C, and as the temperature increased up to 75 °C, the selectivity and flux decreased. It was found that with the addition of PVCA to PDMS, the mechanical and thermal stability improved by more than 25% and 6%, respectively. The results were positive with the combination of PVCA and PDMS, in which lead to the development of a homogeneous dense film structure [119]. Likewise, PDMS-graft copolyimides were synthesized through polycondensation followed by chemical imidization to investigate the effects of the PDMS segment, and then the copolymide membranes were prepared by the solvent-casting method. The gas permeability coefficients of the copolymer membranes increased with the increase in the length of the PDMS segment, but they decreased after heat treatment at 200 °C. In addition, high gas permeability caused by the continuous phase of the PDMS flexible domain, along with improved pervaporation when it requires efficient removal of VOCs from aqueous mixtures, was verified. PDMS-grafted copolymer membranes showed efficiency in removing toxic organic components from wastewater due to an improved pervaporation technique [120]. Additionally, another pervaporation membrane composed of SiO_2_/PDMS/PVDF was manufactured by the dynamic negative pressure method and showed a significant improvement of more than 40% in the contact angle with the surface compared to the PVDF membrane alone. The permeation of phenol into water also increased. The SiO_2_ dispersed in the PDMS solution improved mechanical strength and phenol recovery, which strongly suggests the removal of phenols in wastewater from coal gasification [121].

With the aim of making the pervaporation process more efficient and producing it on a large scale, a non-porous PDMS composite membrane with surface standardization in two stages was manufactured. First, the PVDF substrate was patterned in two layers using phase separation micro-molding followed by modifying dipping precipitation, and then, the PDMS solution was prepared with different types of crosslinking agents that were cast onto the patterned substrate prepared as a selective layer. The permeation of the standardized membrane with the crosslinking agent TEOS showed a larger pattern size, generally more than 2 times higher than the non-standardized one, while with VTES and p-TTES, they improved the polymer stiffness; thus, it is an effective way to improve the pervaporation flow. Micropatterned PDMS composite membranes show great potential in large-scale industrial application for bioethanol recovery [59].

**Figure 5:**
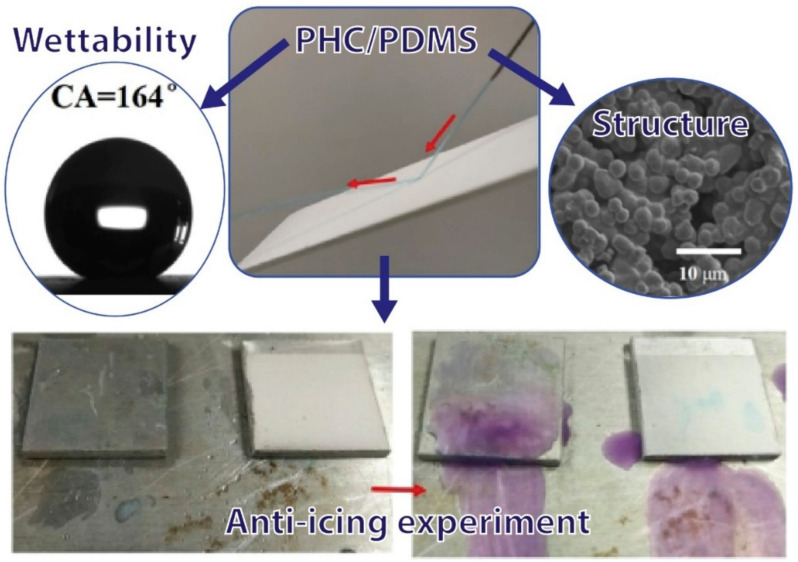
Using PHC/PDMS blends for anti-ice application, adapted from [122].

In the investigation of solutions to ice accumulation on surfaces, superhydrophobic coatings using crosslinked PHC microspheres that have been combined with adhesive PDMS by a single-step precipitation polymerization process have been presented. The coated surface showed a high contact angle and good mechanical durability due to micro/nanoscale structure, as well as self-cleaning and excellent water and ice repellent properties at low temperatures. Its applications are promising and efficient for anti-freeze coatings on external structures, as shown Figure 5 [122].

In several fields of application in biomedicine, PDMS stands as a biocompatible, non-toxic, and transparent material with good thermal and mechanical properties, considering the low cost of manufacturing and raw material. Elastomer PSUs were prepared from amino propyl-terminated PDMS, H12MDI, and APTMDS by a two-step polyaddition route. The PSU films showed high transparency, above 90%, in the visible region, and a low elastic modulus, and the hysteresis values decreased from 32 to 2% in the tenth cycle, and the soft segment refractive index was increased through the incorporation of 14 mol% of methyl-phenyl-siloxane. These results reveal that it is possible to use PSU films to replace the natural human lens after cataract surgery [123].

### 3.5. Other Additions

Another option is emerging in transforming surfaces of different types of substrates into super hydrophobic surfaces using the phase separation method. PDMS is used as a binder, tetrahydrofuran (THF) as a solvent, and water as a non-solvent. The modified fabric showed good self-cleaning ability, antifouling, and an excellent super-hydrophobic property, with a contact angle above 150° and a slip angle below 10°, which are ideal conditions for oil–water separation devices. The advantages of this method are that there is no addition of nanoparticles, thus leaving no marks and no change in tissue color. The easy synthesis method has wide application potential for the fabrication of super hydrophobic surfaces [124].

Polyethylene glycol-blended polydimethylsiloxane elastomeric films were manufactured by a simple mixing process followed by mechanical agitation. The elastomeric films showed an increase in the degree of swelling as the amount of PEG increased, and the young modulus decreased with an increase in the amount of PEG. Although the effect of PEG on PDMS crosslinking deteriorates the mechanical properties of the material, it can be considered positive in terms of increasing hydrophilicity [83].

**Figure 6:**
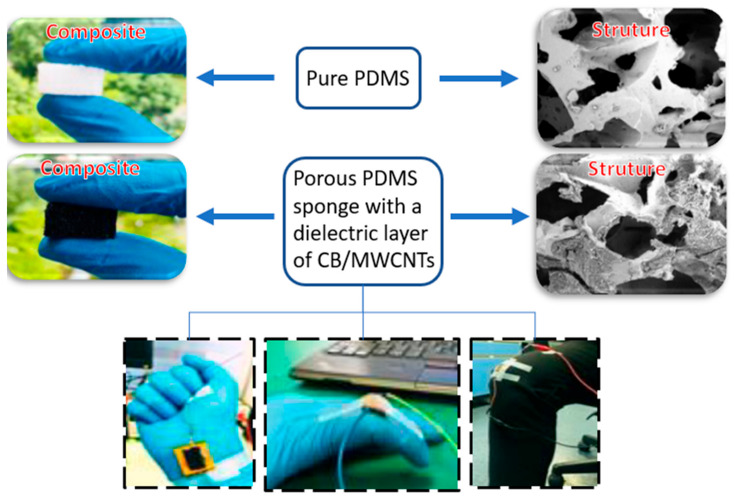
3D porous sponge made of PDMS, carbon black and carbon nanotube for application in monitoring physiological signals, breathing, gripping movements, and heart rate, adapted from [125].

Flexible tactile sensors based on three-dimensional (3D) porous conductive composites were designed with a homogeneous synergistic conductive network of carbon black (CB) and carbon nanotube (MWCNTs) single-dip coating on a polydimethylsiloxane (PDMS) sponge skeleton, as shown Figure 6. The 3D porous structure with hybrid conductive networks of CB/MWCNTs exhibited superior elasticity and excellent electrical characterization under external compression. This piezoresistive tactile sensor exhibited high sensitivity (15 kPa^−1^), fast response time (100 ms), and the ability to detect small and large compressive deformations, as well as mechanical deformability and stability over 1000 cycles. The piezoresistive sensor has been used successfully in monitoring human physiological signals including finger heart rate, pulses, knee flexion, breathing, and finger gripping movements. The highly sensitive piezo-resistive sensor indicates great potential for applications in robotic-assisted surgery systems, human–machine interfaces, and low-cost wearable health electronics [125].

Membranes composed of polydimethylsiloxane combined with other additives have been studied in the most diverse everyday applications. The PDMS-D2HPA compound forms a gel layer on the outer surface of PVDF ultrafiltration hollow fibers through the application of a gallification technique to a new gel extraction membrane (EGM). Optimal extraction efficiency and EGM stability were achieved. The composite membrane showed more improvement in long-term operational stability than EGM flux attenuation, which was only 34% after 120 h, whereas the conventional SLM was 100% after 45 h. The advantages are evident, as EGM shows greater flow and operational stability than the conventional SLM process [126]. However, unlike the electrospinned PVDF layer in PDMS-coated membranes, the electrospinned PDMS/PMMA or PDMS/PMMA/TPU membranes can be applied directly in the separation of wastewater from saline phenol with a better mass transfer coefficient [15].

Another work related to the selection of liquids consists of a porous hydrophobic sponge made of polydimethylsiloxane incorporating a small amount of graphene on the PDMS sponge skeleton surface through a process of mold surface transfer incorporation. The graphene-embedded PDMS sponge showed improved elasticity and durability and required less time to absorb the same amount of oil. However, robust and elastic mechanical sponges can be produced for direct application in oil–water separation in an underwater environment with less absorption time [127].

With the strong demand for superhydrophobic and self-cleaning surfaces, coatings containing PDMS and APTES were manufactured by a simple immersion process followed by room temperature curing. The coatings exhibited high contact angle and high transparency in the visible region, followed by excellent self-cleaning properties indoors and outdoors. In addition, the hybrid coating exhibited excellent antifogging behavior after prolonged exposure to mist and also presented good drop impact durability when applied outdoors [128]. In order to improve surfaces for superhydrophobic and self-cleaning properties, the PDMS-modified PU/Al coating with a smooth surface was prepared using PDMS-modified PU and Al powder flakes as a resin matrix and functional pigment. However, when the PDMS-modified PU/Al coating was modified by nano-SiO_2_, it presented a distinct micro-nano mastoid structure that was formed on the surface, allowing it to reach a contact angle above 150° and a slip angle of less than 10°, thus achieving excellent self-cleaning performance and super-hydrophobicity [129].

Crosslinked membranes were fabricated by introducing LCs into a PDMS matrix using the preferred crosslinking method. The crosslinked membranes produced showed increased mechanical properties compared to pure PDMS membranes. PDMS/LC membranes also showed better membrane formation capacity, a lower hemolysis rate, lower platelet adhesion, and more favorable anticoagulant properties. Furthermore, the mechanical properties and blood compatibility of the membrane may be increased due to the introduction of cholesteric liquid crystals [19].

Surfaces with charge density, roughness, and morphology have a strong influence on the interaction of biomaterials and cells. A hybrid coating with hierarchical surface structures consisting of PDMS and tantalum oxide was fabricated using a simple one-pot, sol-gel-based method. The hybrid coatings showed structures with a combination of micron/submicron and nanoscale characteristics. Structures can be tailored from porous multilayer to single-layer films, depending on the concentration of tantalum oxide. The coatings showed good fibroblast adhesion and cell proliferation in a demonstration of their ability to modulate cell functions. This study demonstrated the coatings’ potential application in the biomedical field to modulate cellular responses and improve implant performance [82].

Another field of application for PDMS is in a multifunctional dressing material, introducing a series of reduced graphene oxide (rGO) sheet contents into the PDMS matrix. The porous membrane was fabricated using the solvent evaporation-induced phase separation technique. The high porosity of the rGO-PDMS membrane on the lower surface is beneficial for cell adhesion and proliferation, and the small pores on the upper surface can prevent excessive water loss in wounds. The addition of rGO blades to PDMS improved mechanical strength, and it can be applied to dressings under high tension. The rGO-PDMS composite membrane showed an increase of 35.33% in mechanical strength and 34.38% in elastic modulus. The membranes also showed the inhibition of bacterial growth and remarkably accelerated wound healing through increased re-epithelialization and the formation of granulation tissue. Therefore, it is a promising material that can be considered a multifunctional dressing [10].

## 4. Results Summary

Table 1 describes the main applications of PDMS mixed with other different types of materials, allowing for the direct and indirect analysis and comparison of some properties, such as tensile strength (TS), ultimate tensile strength (UTS), water contact angle (WCA) and sliding angle (SA). However, there is a summary of what was written in the body of the article discussing some mechanical, optical, and wettability properties.

According to the results presented in the table, PDMS composites modify the mechanical, thermal, and surface wettability properties of PDMS. The additive-type studies by Zieh Pan et al. [127], Xiashi Ren et al. [126] and Gao Shouwei et al. [124] showed that addition composites help to increase the surface hydrophobicity of PDMS sponges and membranes, thus improving the effectiveness for filtering oil in water and extracting heavy ions. Another study by A. Syafiq et al. also modified the surface of PDMS to increase hydrophobicity and fabricate a self-cleaning glass substrate [128]. The works by Wei Qian et al. [10], Huaxin Rao et al. [19], and Long-Fei Ren et al. [15] sought to improve the mechanical properties of PDMS by adding reduced graphene oxide (rGO), liquid crystal (LC), and PMMA sheets for different applications such as biomedical in skin dressing and its direct application in the separation of wastewater. The additive-type PDMS composites presented seek not only to improve mechanical properties and increase surface hydrophobicity, but also to reduce costs, proposing efficient and simple operation and manufacturing methods.

Some other types of materials reported are polymeric nanocomposites. Amir M. Nazari et al. [132], with the objective of manufacturing thin films to be applied in microfluidic devices, reinforced PDMS with nanoclay platelets and, by increasing the nanoclay content, improved the elasticity of PDMS but decreased the shear strength. Yanbing Luo et al. [131] used tetraethoxysilane (TEOS) nanocomposites to impart hydrophobic properties to sandstone and thus preserve it. In general, nanocomposites have physical properties superior to conventional composites in terms of strength, rigidity, thermal, and oxidative stability [132].

The vast majority of studies with nanoparticles were carried out to increase the hydrophobicity of PDMS [97,99,101,129,133], transforming the surfaces of coatings and sponges into super-hydrophobic surfaces, i.e., the angle of contact with water is greater than 150°. These are used for different applications such as self-cleaning surfaces, low infrared emissivity, and the adsorption (separation) of oil in water. However, in some studies [82,102] the hydrophobicity of PDMS was maintained, changing only the roughness [82] and its applications in anti-corrosive coatings [102] and in implants [82]. In a general context, for some applications the disadvantage of composites with nanoparticles is the loss of transparency. Similarly, studies carried out with particles changed the hydrophobicity of PDMS, increasing its value. Shiquan Hang [122] used poly [hexeafluorobisphenol A-*co*-cyclotriphosphazene] (PHC) to create superhydrophobic coatings with an angle of contact with water of 164° in order to produce a self-cleaning and anti-freezing surface with mechanical properties and durability in aggressive environments. However, this type of composite is not suitable for applications where the transparency of PDMS is required.

PDMS composites with different types of waxes mostly alter the hydrophobic, transparency, and mechanical properties. One of the characteristics so far unnoticed in previous studies and observed in wax composites with PDMS is the possibility to control the transparency with increasing temperatures. In works that use paraffin, the composite films at room temperature remain opaque; however, as the temperature increases, the composite becomes transparent, reaching 99% transparency [43], in addition to the use of paraffin, which alters the hydrophobicity of PDMS, making it super-hydrophobic [110]. Another concern demonstrated with this type of composite is the use of biocompatible materials such as carnauba [116], so that the biocompatibility characteristics of PDMS are not changed, which enables the use of the composite in biomedical applications.

Finally, fiber composites with PDMS matrix reinforce the mechanical properties of PDMS Hao-Yang Mi [81] used silica fibers that increased the tensile modulus and strength by 140% and 94%, respectively, with a 20% loss of transparency. We can also note that this type of composite can improve thermal properties; as in the composite with graphene foam, short carbon fibers, and PDMS, this composite improved thermal conductivity by 41%, tensile strength by 52%, and Young’s modulus by 71% [87] when compared to pure PDMS.

## 5. Conclusions

PDMS has become a widely used material in many research fields, and its range of applications increases every year. This increase is reflected in the large number of works that have been studying and modifying this material, resulting in tailored properties for extremely specific purposes. This review provides an outlook of how PDMS composites could have their main properties improved, such as their mechanical, electrical, and optical features, opening up new avenues and applications in various fields of engineering. The next generation of PDMS composite materials should focus on improving and standardizing the manufacturing of PDMS mixed with other materials, such as waxes, and seek efficient processes that facilitate the manufacturing process at a low cost, which would allow for the replacement of conventional processes and large-scale engineering application. Additionally, PDMS can be widely explored in new emergent technologies, as in the field of transparent films for photovoltaic panels, which continues to be a future trend for sustainable and renewable energy.

## Figures and Tables

**Table 1:** Summary of mechanical, optical, and wettability properties of PDMS combined with other materials.

Type	Description	Reinforcement	Property	Value/Change	References
Additive	Membrane	PDMS-PEG1	Young’s modulus TS	Decreased 22.0% Decreased 6.0%	[83]
Additive	Sponge	Graphene/PDMS	WCA	128.9 ± 2.3°	[127]
Additive	Membrane	PDMS-D2EHPA	WCA	102.0 ± 2.0°	[126]
Additive	Sponge	THF as the solvent	WCA	155.0 ± 0.6°	[124]
Additive	Membrane	PDMS/PMMA	TS at break Elongation at break	1.7 MPa 60.0%	[15]
Additive	Membrane	rGO-PDMS	TS Young’s Modulus	Increased 35.33% Increased 34.38%	[10]
Additive	Membrane	PDMS/LC	Strain at break Stress at break Elastic modulus	Increased 7.0% Increased 78.0% 4.7 MPa	[19]
Additive	Coating	PDMS/APTES	WCA Transparency	103.9° 91.2%	[128]
Blend	Membrane	PDMS/PVCA	UTS Elastic modulus	133.7 MPa 2400 MPa	[119]
Blend	Membrane	PIS6	WCA Young’s Modulus TS Elongation at break	99.2° 400 MPa 20 MPa 100%	[120]
Blend	Characterization	PSU-3T	Young’s Modulus UTS Elongation at break	5.5 MPa 6.0 MPa 880%	[130]
Nanocomposite	Coating	PDMS/TEOS	WCA WVP	130.0° 7.9 × 10^−8^ $\frac{g}{\mathrm{msPa}}$	[131]
Nanocomposite	Films	PDMS-clay	Elastic Modulus	1.5 MPa	[132]
Nanoparticle	Coating	Tantalum oxide/PDMS	WCA	110.0°	[82]
Nanoparticle	Coating	PDMS/PU-Al and SiO_2_	WCA SA	151.5° 9.0°	[129]
Nanoparticle	Coating	PDMS/Spray-coated CNP	WCA	167.0°	[133]
Nanoparticle	Sponge	PDMS/SiO_2_/WS_2_	WCA Separation Efficiency	158.8 ± 1.4° 99.8%	[97]
Nanoparticle	Coating	SiO_2_/PDMS and Beeswax	WCA SA	154.6° 5.0°	[98]
Nanoparticle	Coating	PDMS/TiO_2_	WCA Properties	102.0° Improved the anticorrosion	[102]
Nanoparticle	MechanicalProperties	MQ resin in silica sol and V-PDMS	Young’s modulus TS	0.2 MPa 1.9 MPa	[134]
Nanoparticle	Coating	PDMS/TiO_2_	WCA SA Separation Efficiency Properties	158.0° 5.0° Oil/water 99.5% Improved Abrasion Resistant	[100]
Particle	Coating	PDMS/PHC	WCA SA Properties	164.0° 3.7° Improved Mechanical Durability	[122]
Particle	Membrane	PDMS/SiO_2_/PVDF	WCA Elongation at break	131.8° 158.0%	[121]
Particles	Membrane	PDMS-silicate-1	WCA	135.2°	[50]
Wax	Coating	PDMS-MCNTs-Beeswax	WCA SA	158.3° 1.4°	[115]
Wax	Coating	Carnauba wax/PDMS-paper	WCA SA	169.0° 3.0°	[116]
Wax	MultifunctionalMaterial	PDMS/Paraffin	Transparency	80%	[108]
Wax	MultifunctionalMaterial	PDMS/Paraffin	Transparency	85.5%	[109]
Wax	MultifunctionalMaterial	P-PDMS	Transparency	~94.0%	[112]
Wax	MultifunctionalMaterial	PDMS/Paraffin	Transparency	85.0%	[114]
Wax	Coating	Carnauba wax/PDMS	WCA SA	162.0° 10.0°	[117]
Wax	Coating	PDMS/Paraffin	WCA Separation Efficiency	156.7° Diesel oil/water 95%	[110]
Wax	Mechanical Properties	PDMS/Paraffin	TS Transparency	2 MPa ~99%	[107]
Wax	MechanicalProperties	PDMS/Beeswax	WCA TS Hardness Transparency	129.3° 1.1 MPa 28 [Shore A] 71%	[75]
Wax	MechanicalProperties	PDMS/Paraffin	WCA TS Hardness Transparency	141.9° 2.6 MPa 33.2 [Shore A] 72%	[75]
Fiber	MechanicalProperties	Poliacrilonitrila-graft-PDMS	Young’s modulus Tensile strength	Increased 56% Increased 60% non-woven	[86]
Fiber	MechanicalProperties	graphene foam/PDMS	Young’s modulus Tensile strength	Increased 71% Increased 52%	[87]
Fiber	Mechanical Properties	Silica continuous/PDMS	Maximum strainTensile Strength	Increased 94% Increased 140%	[81]
